# Current Status of Health and Social Services for Children with Autism in Iran: Parents’ Perspectives

**Published:** 2019-01

**Authors:** Hadi Zarafshan, Mohammad Reza Mohammadi, Farid Abolhassani, Seyed Abbas Motevalian, Neda Sepasi, Vandad Sharifi

**Affiliations:** 1 Psychiatry and Psychology Research Center, Tehran University of Medical Sciences, Tehran, Iran.; 2 National Institute of Health Research, Tehran University of Medical Sciences, Tehran, Iran.; 3 Department of Epidemiology, School of Public Health, Iran University of Medical Sciences, Tehran, Iran.; 4 Department of Psychiatry, Tehran University of Medical Sciences, Tehran, Iran.

**Keywords:** *Autism*, *Iran*, *Parent*, *Qualitative Study*, *Services*

## Abstract

**Objective:** The present study is a part of a larger study that aimed at developing a comprehensive model of service delivery for individuals with autism in Iran based on the local needs and characteristics. In this study, the status of the services provided to those with autism in Iran was investigated based on the perspectives of parents of children with autism.

**Method** **:** A semi-structured interview was used to qualitatively investigate the status of the services with regards to autism in Iran based on the perspectives of parents of children with autism.

**Results: **This study revealed several barriers, including shortness of eligible professionals, difficulty in access to care for families, high costs, and lack of formal and informal supports, in providing services to those with autism in Iran. Early detection and diagnosis are of prime importance. We also need to train our specialists to diagnose autism based on the standard protocols and tests.

**Conclusion: **In sum, we need a comprehensive program that involves different sectors in charge of health and education in Iran.

Autism is one of the most complex childhood psychiatric disorders whose effects usually continue through the life (1-3). People with autism need support to attain their abilities in different levels (4-6). The increase in autism prevalence (7, 8) has made it an important public health concern and has attracted the attention of the policymakers to provide the necessary services for these patients and their families (9, 10).

Pervious researches have shown that people with autism and their families are in greater risk to have unmet needs and lack of access to health care services (6). However, due to the complexity of their condition, they need more special and general health and educational services than the typically developing individuals or those with other disorders (11, 12). Those with autism need more resources and impose high costs on the society (13). 

A recent study reviewed the direct and indirect costs of patients with autism in the United States and the United Kingdom and found that the lifetime cost of an individual with autism and intellectual disability was 2.4 million dollars and 2.2 million dollars in these countries, respectively (14).

The prevalence of autism in Iran is close to its global prevalence (7, 15 and 16). Based on the national census in 2016, the population of Iran is more than 79 million. If we consider the prevalence of 1% for autism during lifetime, there are close to 790,000 individuals with autism in Iran who may need help and support at different levels.

Given that we are faced with a huge number of affected individuals with autism in Iran and considering the complex and high unmet needs of this group and their families, it is necessary to design and develop evidence-based services that meet their needs. To achieve this goal, we need to have a clear picture of the status of the available health and support services for this population. 

This study was a part of larger study that aimed at developing a comprehensive model of service delivery for individuals with autism in Iran based on the local needs and characteristics. Because parents are the main source of care for people with autism, they can provide valuable information about the existing services and characteristics of a program that fits the needs of people with autism. Thus, in this study, semi-structured interviews were used to qualitatively investigate the status of the services provided to individuals with autism in Iran based on the perspectives of parents of children with autism.

## Materials and Methods

In this qualitative study, semi-structured interviews were used to explore the status of those with autism in Iran from their parents’ perspective. 

The interviews were conducted after obtaining approval from the Ethics Committee of Tehran University of Medical Sciences. Interviewees were selected conveniently among the parents of children with autism, who were receiving rehabilitation services from an NGO (Omid Bavar) and a private child psychiatry clinic in Tehran, the capital of Iran. Because these centers provide services to families residing in Tehran and other small cities around it, they could be, to some extent, representative of those families with middle class social status in Iran. Participants included 10 parents of children with autism.

Before conducting the interview, the purpose of the study was explained to the parents and oral consent was obtained. Interview sessions were face-to-face and conducted when children were participating in training or educational classes. Each interview was conducted with 1 parent and lasted up to 60 minutes. 

One of the investigators (HZ) conducted all the interviews, and 2 pilot interviews were done before the main interviews.

The study team members designed the semi-structured interviews based on the study goals and the literature. The interview included questions about the following items: general information about the family members (Would you please tell me about your family? How many people are there in the family? How old are they?), initial symptoms of their children and worries of parents (What was your first worry about your child? How old was she/he at that time?), contact with a specialist (first or regular) (To which specialist did you refer to share your worries about your child?), diagnostic procedures (What did the specialist/s do to evaluate your child? Which examinations did they use? How did they inform you about your child’s autism?), using special training centers (After receiving the diagnosis, what kind of training centers contacted did you contact?), training courses (What kinds of services has your family used for your children with autism up to now?), burden on the family (How much is your monthly family income? How much is your child’s monthly cost? What problems have you faced because of your child’s autism?), possible causes of autism from parents’ perspective (What do you think about causes of autism? What about your family members?), and parents’ experiences in their journey (How do you feel about having a child with autism? What are your worries?).

Data Collection and Analysis

All the interviews were voice recorded and transcribed by one of the team members. Then, the transcripts were read carefully and checked by the interviewer to explore any possible problems. The framework approach was used to analyze the materials (17). The interviews were analyzed by the members of the study team in group sessions.

Some information that the respondents did not want to be published was not used and instead other information was used anonymously.

***Ethics***

All procedures performed in studies involving human participants were in accordance with the ethical standards of the institutional and/or national research committee and with the 1964 Helsinki declaration and its later amendments or comparable ethical standards.

Informed Consent: Informed consent was obtained from all individual participants in the study.

## Results

***General Information about the Family Members***

All the respondents were mothers with a child with autism spectrum disorder (ten mothers). The children were 5 (50%) boys and 5 (50%) girls. Of them, six were diagnosed as having autism (60%), three as having Asperger disorder (30%), and one(10 %) as having pervasive developmental disorder-not otherwise specified. They all lived in Tehran and nearby towns (Varamin, Pakdash, Shahriar, and Karaj). The mean age of children was 91.7 months (range: 40 -173). The respondents’ (mothers) mean age was 36.3 years (range: 24-46).

***Initial Symptoms, Contact with a Specialist and Receiving Diagnosis***

Nearly all the respondents (9 of 10, 90%) said that their first concern was their children’s communication problem. Communication problems were lack of or delay in language development and eye contact, mostly in response to their name. One of them had irritability and hyperactivity in early years of life. 

 “My daughter didn’t communicate with us. In comparison with other children her age, she did not point to anything. When she wanted something, she just cried.” 

 “My son didn’t speak at 2.5 years old. He did not speak at all; he just made meaningless sounds. When he wanted something, he took my hand and took me near it.”

“My daughter was irritable from the beginning (early infancy). She has been speaking fluently since two years old, but she was always screaming and shouting.”

“When my son was one year old, he didn’t react when we called his name. We suspected a hearing problem.”

The mean age of the first concerns was 26.4 months old (range: 12-41) among our sample. Three of the mothers (30%) reported that their children had showed their problems after a typical period of development. They usually had shown regression in their abilities in ages 24 and 36 months.

“He seemed a typically healthy baby at first (early toddlerhood) and had no problem until his 3.5 years old. On the New Year’s Eve, we noticed that he did not play and speak any more, and just sat and stared out into space. He did not play with his toys. He would have told us that he had to pee before, but did not say it anymore. He was a typically healthy child before that. He has been like this since 2 or 3 days before the New Year. When we went to visit our family, they understood that my son had changed. After a while, he began to cry and laugh for no reason; he was nervous and threw objects to the walls.”

After the parents noticed the first abnormal symptoms, they shared their worries with family members such as grandmothers and grandfathers, aunts, and other family members who had a child previously. The first common signs were communication problems (usually language delay), and when parents shared it with other family members, they usually responded that it was normal because language delay runs in the family. 

 “When I shared my worries and my child’s problems with my parents, they told me that my daughter had no problems and I should not label my child …“

 “In fact, given that my husband has had language delay, he started to speak at the age of 4, when we shared our worries (language delay in our children) with our family, they told us that we should not be worried because my husband had the same problem when he was a kid but now he can speak perfectly.” 

After a while, when the family members noticed that the child problems do not get better gradually, they agreed to take the child to a specialist.

“My son was 32 months old when my father-in-law said that he thought my son had a serious problem and that we should take him to a specialist.”

At first, the parents usually contacted a pediatrician, internist, or speech and language pathologist. The mean age of the first contact to a specialist was 36 months (range: 12-60). At the first visit, specialists usually suspected a hearing problem and sent children for Auditory Brainstem Response ABR test. When they found no hearing problems, they said that the child would get better and that there was no need for any intervention, suggesting that the child only had language delay and needed speech therapy.

“The first time, I made an appointment with an internal specialist; I took my daughter to his office when she had a cold. I told him we should shout to see her response. He told us she may have a hearing problem. He sent us to ABR test, but her ABR test result was normal.”

“We brought our son to a pediatrician office for monthly check-up. I expressed my concerns about my child’s behavior to her. She knocked the table and my son did not turn his head. She told us your child has a hearing problem. She referred us to an audiologist. The result of the test showed that he had no hearing problems.”

 “She was a speech and language pathologist. She did not diagnose autism at all. She told me there was no problem and that boys usually have language delay; she absolutely wasted our time for a year.” 

“When my daughter was 2 years old, I regularly brought her to the health center for developmental check-up. They told me she is perfect. I was complaining that she could not speak, but every time they told me not to worry, she has time to speak until she reaches 3 years old.” 

Those parents, who had contacted a child psychiatrist initially had received correct diagnosis in the first visit (30%). Other respondents said that when they found something was still wrong, they contacted another specialist, usually a child psychiatrist or a neurologist, and received autism diagnosis for their child. Most of the children were diagnosed by a child psychiatrist for the first time (60%). Two of the children (20%) were diagnosed by a child neurologist, and 2 (20%) by a psychologist. The mean age at the first diagnosis was 51.4 months old (range: 18-120). 

As the respondents revealed, specialists did not use any specific protocols or tests to diagnose autism. Only the child neurologist used medical tests, such as EEG and metabolic examination. They diagnosed autism in a brief session (less than 30 minutes on average) based on their observation and some quick questions.

“My son was sleeping in my arms. The doctor asked some questions. He asked me whether my child responded to his name, or did he do as I tell him? And then he referred me to an autism center.”

“Right after we entered the doctor’s office and the door was closed, my son began to scream. He cried and shouted like a crazy person. She could not calm my son down. At the end, she told me she thought my son had autism.”

Based on parents’ experience, none of the specialists, who diagnosed their children for the first time, provided an adequate explanation of the problem and did not prepare them to accept this issue and the intervention process.

“I took my son to a psychiatric hospital; she (psychiatrist) visited him there. During the visit, she just talked to her assistant; they used English terms that I did not understand at all. She did not treat us well. After the visit, I cried all the way to my home. I didn’t know what to do.”

“I took my child to a doctor. He asked me if my child responded to his name; I said he did not. He immediately told me that my child has autism.”

Four of the specialists (40%) referred the family to a special center to receive intervention. Other specialists (60%) just said that the child needed intervention. 

***Service Use and Availability***

Parents had used a wide range of services for their children. At the beginning, when their children were very young, most families had used private centers, clinics, and organizations services. These services included occupational therapy, speech therapy, and applied behavioral analysis (ABA). Some families had used special day care centers for children with autism that operate under the supervision of Social Welfare Organization. When their children got older (around 6 years old), some of the parents (30%) registered their children in special schools for children with autism that operate under the supervision of Special Education Organization. Four of the families (40%) had used special trainers in their home for a while in addition to other services (between 6 months to two years). Some university-affiliated clinics provide rehabilitation services (Three of the families had used this service for a while.).

The duration of using intervention ranged from 1 year (for newly diagnosed children) up to 10 years (for older children), with more intensity in the early stages of using the services. Based on the interviewees’ responses, on average, they have used between 5 to 15 one-to-one training hours per week for their children (including ABA program, occupational therapy, and speech therapy).

All the families said that they used psychiatric medication for their children. Risperidone and Ritalin were the most common medications; all children have used these medications at least for a short period.

Most families (90%) preferred services that were prepared by private centers, clinics, and organizations. They mentioned that the staffs of the private sector are usually more skillful, but their services are expensive. Moreover, families could choose their desired services in private services freely.

Families mentioned some barriers to use preferred services for their children; one of them was the cost of the services.

“In previous centers, we had to pay too much and my husband couldn’t afford it anymore.”

“I should mention that the services were highly expensive; I had to spend all my salary.”

“If I had not gotten discounts from this center, I would have had to cancel the classes.”

Another obstacle for families is availability of the services. They should spend much time and go long distances to access the services.

“There is no place near our home. Each day, I have to spend 2 hours to get to the clinic; it’s very hard for me.”

“I come from a very far distance, I wish there was a good center near us.”

“I’m coming from Pakdasht, it takes me 6 hours each time to get here; no similar services are offered in our town. There are only speech therapy service? in the municipality.” 

Four mothers said that they have used some kinds of traditional interventions like talisman, pigeon eggs, and energy therapy.

***Impact on the Family***

Based on the family reports, on average, the family monthly income was 17,300,000 Rials (equivalent to 533 USD), ranging from 9,000,000 (equivalent to 277 USD) to 25,000,000 Rials (equivalent to 771 USD), and child’s monthly training costs was 7 400 000 Rials (equivalent to 228 USD), ranging from 5,000,000 (equivalent to 154 USD) to 10 000 000 Rials (equivalent to 308 USD). It means that near 43% of the family income was spent on educational services for this member of the family.

In addition to the financial burdens, having a child with autism caused several personal and interpersonal problems for the family members. Based on the interviews’ analysis, the problems were as follow: Parents did not have enough time for each other (100% of the respondents); spouse conflicts (30% of the respondents); they did not have enough time for their typically developing children (100% of families who had another child); mothers left their job (30% of the respondents); problems in social and leisure activities because of their child’s behavior (100% of the respondents); anxiety and depression among parents (60% of the respondents); and extra working times to cover child’s expenditure (100%).

 “I really don’t know what I should say, but whoever is a parent of an autistic child is faced with very serious challenges in life. For example, if I want to go anywhere, my husband should be at home and take care of our son and vice versa.” 

 “It has made us very isolated, we can’t go to parties, we don’t have much time for each other (mother and father), and my husband has to work from early morning to late at night.” 

 “When my daughter became sick, I was so preoccupied that I forgot my older daughters; I didn’t have time for them and they got involved in relationships with older men.” 

“For example, I messed up for a long time, and it caused conflicts between my spouse and me; he told me that I only pay attention to our son.”

***Supports***

All respondents reveal that there is no adequate governmental financial support. Two families said that they have registered their children in Social Welfare Organization and received financial support. One of them said that her child receives a monthly support of 480,000 Rials (equivalent to 15 USD) and another one said that they received 6,000,000 Rials (equivalent to 185 USD) for a year. One mother said, “I referred to Social Welfare Organization for my daughter, but they said my daughter does not have serious problems and that they can’t help me.” 

Other families did not want to register their children in Social Welfare Organization due to the long process and mostly for the stigma of registration.

“If I had registered my son in Social Welfare Organization, they would have labeled him as a mentally ill person and I did not want that …, moreover, their financial support was not much.”

There is no public insurance coverage for rehabilitation services in Iran. Only some big organizations such as ministries, banks, and military services have supplementary insurances that cover parts of mental health and rehabilitation services cost. Among our sample, 2 (20%) had supplementary insurances that covered parts of their expenditures for their children.

“…One of the barriers for us is that the government does not have insurance coverage for rehabilitation services for these patients. It would have been wonderful for these children if the government supported these families ….”

Four families (40%) said that they received support from charities and that without their support, they could not continue the training programs.

Most parents had to care for their children as there were no support offered to them by the government or their families. Only 2 of them (20%) said that their families helped them if they needed to go somewhere without their children. There are no specific centers to help families or look after their child for a short time.

“... I can’t understand why when others (relatives) notice your child has problems, they do not support you, but they do that for typically developing children easily ….”

***Current and Future Needs and Worries***

Families declared different kinds of needs. They said that there should be a supporting system to prepare the family to cope with this issue.

“I wanted them to support us when we realized the problem. My other son was 11, and we could not give enough attention to him because we had no time for him. Our life was full of conflict.”

The families emphasized the need to raise awareness among families about typical development and receiving appropriate help on time.

“The community should do something to increase awareness among families to avoid delay in the treatment process.”

All families revealed they needed to be educated about their child’s condition and how to treat them.

“An important factor that can motivate families to follow the treatment process is that the clinicians explain children’s problem in simple language for the families.”

“I think there should be a counselor in this center to guide us how to treat our children because we have an important role in their treatment.”

Availability of high quality rehabilitation services and schools is another need of the families. Parents also revealed that they need short- and long- term residential centers.

“There should be some places or centers to keep the child for a while if the parents want to spend time together. It means we should consider other family members’ needs. If we have enough energy, we can take care of our children better.”

Families believed that their children should not be separated from their typically developing peers and community.

 “Why these children should be separated from those who go to regular schools; why they shouldn’t be with typically developing children? The community should accept and tolerate these children. We should raise public awareness.”

Planning for leisure time programs was another issue that the parents emphasized.

 “I’d like sport classes for our children. I wish he could be trained to swim and play some instruments.”

“I think sport classes are good for my son, but no such classes are there for these children.”

Parents who had older children emphasized the need for providing training to the children for puberty and preparing them for job and independent life skills.

 “No one educated my daughter about puberty issues; I did it myself, but I do not know if it was effective.”

 “There should be some places that my son could acquire skills for later job acquisition and independent life skills.”

We asked parents about their worries for their child’s future life. They said that they are worried about their child living independently and about their child’s life after their death. Other issues were legal problems about their children’s inheritance rights, military service, and guardianship.

“I am worried about his lack of ability to handle his life independently.”

“I always think about what would happen to my child after I die? Who cares for our daughter?”

 “We want to move from Iran to somewhere, so there be at least a place that takes care of our son after we die.”

“My son is growing up. He should go to the military service. I don’t know what will happen.”

 “We bought an apartment for our daughter, but she can’t live independently. We are not sure what would happen to her after we die.”

## Discussion

Our study revealed that there are several gaps in providing services to those with autism in Iran. Early detection and diagnosis are of prime importance. Based on our interviews, in all the sample family members, when they had noticed the child’s problem for the first time they referred to a specialist and, in some cases, the specialist had dismissed the problem. Early detection and intervention maximizes outcome in people with autism and decreases future costs and burdens for the family and the society (18). Thus, we need to have some kind of screening or early detection services to detect children who are suspected of having autistic disorder during their early years of life (19). Moreover, we need to increase public awareness about typical development in early years and red flags for autism (19). We also need to train our specialists to diagnose autism based on the standard protocols and tests (19).

Another issue that should be considered is preparing families to cope with their child’s condition and guide them to use proper services after they receive the diagnosis (20). Based on the respondents’ experiences, none of the specialists prepared them for their child’s condition properly and only informed them about the diagnosis and left them alone. The same issue has been reported in previous studies in Iran (21); they indicated that they need to know more about children with autism. We should train our specialists to provide adequate information to the parents and prepare them to cope with such a disappointing issue. Family support groups can also play an important role in helping the families cope with their new condition (20).

 Shortness of qualified special centers and service providers is another obstacle. In Ahmadi et al. study (22), accessing services and professionals was ranked first by Iranian families. We need to design a program to train specialists based on the evidence- based approaches to provide services for people with autism. Given the prevalence of autism and shortness of specialists, this program should consider training people with lower academic degrees and in settings with limited resources (ie, task shifting and task sharing). There should also be governmental planning to increase the number of schools for special children, especially in suburban areas. We can also use the capacity of NGOs and charities to increase services for people with autism.

In addition to the shortness of services, similar to many other countries (14), the cost of services is another problem for the families. Based on our study, on average, 50% of the family income should be spent for training the autistic member of the family. Insurance coverage for rehabilitation and psychological services can reduce family burden (23). In addition, training and using paraprofessionals (24) and family members (25) as a therapist can reduce the cost of interventions.

Having a child with autism imposes burden to the family members (14). Based on our study, in addition to the financial burdens, families with autistic children are faced with several personal and interpersonal obstacles, which are as follow: parents not having enough time for each other; marital conflicts; parents not having enough time for their typically developing children; mothers leaving their job; problems in social and leisure activities because of the child’s behaviors; anxiety and depression among parents; and extra working times to cover child’s expenditure. We need to develop different types of family support programs, respite care programs as a supportive approach, make free time for parents and increase marital quality, and decrease parental stress (26). Creating sibling support groups (27) and training sibling about autism (28) can help typically developing siblings of children with autism to better understand and cope with living with a sibling with autism.

Based on parents’ expressions, other issues should also be considered. We need to establish residential centers specifically designed for people with autism. In developing countries, such as United Kingdom and United States, residential care during adulthood is the main source of autism costs (14). There are different components such as role of family, staff training, program components, and interaction with non-handicapped community that are necessary in designing residential programs (29). Children with autism have minimum interaction with their typically developing peers in Iran. Their rehabilitation services are located in special centers, and only those children with minimum symptoms go to regular schools, but others are educated in schools for children with special needs (30). Inclusion of people with autism in regular schools and other settings with typically developing peers have positive effects on social skills and performance of children with autistic disorders as well as their peers (31). An important issue is that when inclusion programs start early, we will have better outcomes in the future, so the inclusion programs should start in the preschool period (32). 

Other finding was that as children with autism grow up, their needs change to leisure time activities, training for puberty, sexual issues, acquiring job, and independent life skills. Another important issue in adolescent and young adult period is legal problems about their inheritance rights, military service, and guardianship. Based on our interviews, we do not have any predetermined program to teach these skills to people with autism or support their families to overcome these obstacles. Based on the literature, the main factor that affects social outcomes in adulthood is the suitability of educational provisions and access to proper education for later employment and social independence (33). The job placement programs have been effective in job acquisition for people with autism, especially for lower level jobs (34).

## Limitation

The most important limitation of this study was that our sample size was small and limited to Tehran and its nearby cities. Because Iran is a vast country with different cultural and socioeconomic status, our sample may not be representative of all parents with a child with autism in Iran.

## Conclusion

Many issues are overlooked in the field of autism in Iran. Thus, we need a comprehensive program that involves different sectors in charge of health and education in Iran. Early detection and intervention are the most important components of this programs. Another issue is that the existing services are expensive and not available and therefore there is a need for the provision of services that have more coverage.
